# Digital Solutions for Health Services and Systems Management: Narrative Review of Certified Software Features in the Brazilian Market

**DOI:** 10.2196/65281

**Published:** 2024-11-29

**Authors:** Ericles Andrei Bellei, Pedro Rafael Domenighi, Carla Maria Dal Sasso Freitas, Ana Carolina Bertoletti De Marchi

**Affiliations:** 1 Graduate Program in Human Aging, Institute of Health, University of Passo Fundo, Highway BR285, São José, Passo Fundo, 99052-900, Brazil, 55 5433168384; 2 Undergraduate Program in Computer Science, Institute of Technology, University of Passo Fundo, Passo Fundo, Brazil; 3 Institute of Informatics, Federal University of Rio Grande do Sul, Porto Alegre, Brazil

**Keywords:** health services administration, health information management, decision support systems, digital health, Brazil, certified software, features, systems management, health services, interoperability, digital solutions

## Abstract

The paper reviews digital solutions for health services management in Brazil, focusing on certified software features. It reveals the integration of various functionalities in operational, financial, and clinical needs simultaneously, which are critical for enhancing operational efficiency and patient care. This study highlights the integration of critical features like interoperability, compliance management, and data-driven decision support, although advancing innovation and integration remains essential for broader impact.

## Introduction

Digital technologies are crucial components in enhancing the efficacy and quality of health services and systems management [1]. The global health care sector faces mounting pressures from escalating service demands, demographic transitions, and economic constraints, necessitating the integration of digital solutions [2]. This imperative is driven by the need to optimize resource allocation, improve patient outcomes, and streamline operations within an increasingly complex and demanding environment [3]. Health managers, responsible for the continuous operation of intricate systems, require robust and reliable information to make informed decisions that enhance system performance and patient care [4].

The adoption of commercial digital solutions is essential for improving operational efficiencies and patient care [5]. However, a significant gap exists between their potential as described in scientific literature and their performance in real-world settings [6], with off-the-shelf solutions, for instance. This disparity necessitates a review of commercial digital solutions in health care, focusing on their practical application in actual health care environments [7]. This study’s objective is to review commercially available solutions in health care management, with a specific focus on their features and functionalities.

## Methods

### Overview

To understand off-the-shelf digital solutions and their functionalities, this study included identification, screening, extraction, and qualitative synthesis phases. Given the variability in features and configurations across different off-the-shelf solutions, we aimed to establish a standardized baseline by focusing on solutions certified by recognized organizations. In Brazil, the Brazilian Society of Health Informatics (*Sociedade Brasileira de Informática em Saúde*; SBIS) provides such certifications, created in partnership with the Federal Council of Medicine. The certification process evaluates and attests to the quality, security, and privacy aspects of electronic health record (EHR) systems, ensuring compliance with various standards, norms, and best practices. The certification categories include clinic or outpatient services EHR, individual practice EHR, inpatient services EHR, emergency care services EHR, information security solutions, and telehealth or teleconsultation solutions.

From the list of certified systems updated in August 2024, we selected software platforms that held certification in “clinic or outpatient services EHR” and at least 2 additional categories. This criterion was used to identify multimodule solutions or platforms capable of supporting a broader health care system. Ultimately, 7 software platforms were selected from a pool of 20 certified systems. To extract and synthesize their characteristics, we analyzed the SBIS certificates associated with each system, focusing on the specified functionalities and capabilities. Our analysis was guided by the functional requirements theory of software engineering. Subsequently, we visited each system’s official website to gather descriptions, demonstrations, and technical documentation related to functionalities. We manually reviewed all subpages of each website, including user manuals in PDF format, to extract both textual and visual information available for the Brazilian market in Portuguese. The extracted data were consolidated narratively for each system and then clustered into categories and descriptions.

### Ethical Considerations

This study did not involve human subjects or sensitive data and therefore did not require ethics board review or approval.

## Results

The narrative synthesis included 7 systems—SMED, Soul MV, Philips Tasy, SPDATA, Feegow, TI.Clinic, and PEP Unimed—from the SBIS list [8]. Table 1 summarizes the functionalities. Not all systems have all the comparable features. Also, the features listed are aimed at professional managers and not necessarily at clinical practitioners.

**Table 1.** Summary of features from certified software for health services and systems management in the Brazilian market.

Feature category	Description
Service cycle management	It includes scheduling; hospitalization; urgency and emergency; bed management; companion/visitor control; service panel; and indicators such as bed occupancy rate, average waiting time, and readmission rate.
Financial and administrative management	It covers complete financial management, including the administration of multiple institutions, control of accounts payable and receivable, bank reconciliation, invoicing of agreements, tax deductions, financial and accounting reports, and control of cash flow and balance sheets, in addition to indicators.
Asset and equipment management	It involves the registration and monitoring of the institution’s assets, including inventory, preventive and corrective maintenance, and the management of medical equipment and infrastructure.
Cost management	It involves identification and optimization of operating costs, including cost analysis by control centers, materials, services, costing methods, and productivity indicators.
Management of relationship with health maintenance and insurance organizations	It involves complete management of contracts with health operators, including billing, disallowance control, claims, performance of the accredited network, and beneficiary management.
Supply and materials management	It covers the acquisition, inventory control, and traceability of medicines and hospital materials; scheduled purchases; and management of critical supplies in accordance with safety and regulatory standards.
Sterilization and sanitation management	It involves the control of sterilization and sanitation processes, including the management of beds, hospital materials, and layette stock.
Quality and safety indicators	It involves monitoring of critical indicators, including hospital infections, adverse events, and patient safety, in addition to the analysis of deficiencies in hospital processes and identification of root causes.
Human resources management	It involves the control of shift schedules, absenteeism, turnover, and overtime, with a focus on talent retention and operational efficiency.
Environmental management	It involves the monitoring of water consumption, energy, and waste generation, with a focus on sustainability and operational efficiency.
Interoperability and information exchange	It enables the exchange of information with other hospital management systems, including the implementation of standardized rules and integration with regulatory systems such as those from the National Agency of Supplementary Health.
Compliance and audit management	It involves continuous monitoring of compliance with local and international regulations, auditing of internal processes, and automatic reporting for inspections and certifications.
Supplier relationship management	It allows the management of contracts and supplier performance, with contract renewal alerts, quality and punctuality assessment, and supply chain optimization.
Clinical research management	It involves tools to manage clinical trials, including participant recruitment, data collection, protocol monitoring, and results analysis, with compliance with ethical and scientific regulations.
Infrastructure and IT management	It involves centralized control of IT infrastructure, including monitoring of servers, networks, and connected medical devices, as well as cybersecurity management and data backups.

## Discussion

The summary reveals a trend toward integrated systems that address operational, financial, and clinical needs simultaneously. Key features, including interoperability, compliance management, and data-driven decision support, are essential in Brazil’s fragmented health care landscape. Interoperability enables seamless information exchange, crucial for coordinated care, while compliance management ensures adherence to regulatory standards, reducing risks for health care institutions. Data-driven decision support tools allow health care managers to leverage predictive analytics for resource optimization and improved patient outcomes. These features collectively reflect a shift toward holistic, unified platforms capable of meeting modern health care demands. Compared to global standards [9], Brazilian certified systems offer competitive features with room for further innovation, particularly in addressing regulatory issues, costs, and infrastructure limitations that challenge widespread adoption.

As health care institutions navigate an increasingly complex environment, the development of adaptable and secure digital management solutions will be essential [10]. Establishing national interoperability standards could create a more cohesive health care ecosystem. Additionally, investments in infrastructure, standardization, and workforce training—particularly in underserved regions—would broaden digital access across Brazil’s health care system. Addressing these areas will be critical to advancing scalable and impactful digital solutions. Future advancements will rely on integrated systems capable of predictive analytics to improve decision-making, uncover trends, and drive informed strategies systemically.

## Abbreviations

EHR: electronic health record
SBIS: *Sociedade Brasileira de Informática em Saúde* (Brazilian Society of Health Informatics)
